# Supplementary material for: Generalized monodromy method in gauge/gravity duality
Source: Sci Rep. 2022 Jul 14;12:11988. doi: 10.1038/s41598-022-16054-0 (PMC9283430; doi:10.1038/s41598-022-16054-0)
Supplement: Supplementary file 1 — Supplementary Information. [file 41598_2022_16054_MOESM1_ESM.docx]

**Generalized Monodromy Method in Gauge/Gravity Duality**

**Yuanpeng[[1]](#footnote-0)HOU**^1,2,3*^

1. Xinjiang Astronomical Observatory, Chinese Academy of Sciences,150 Science 1-Street, Urumqi 830011, China

2. Kavli Institute for Theoretical Sciences (KITS), University of Chinese Academy of Sciences, Beijing 100190, China

3. School of Astronomy and Space Science, University of Chinese Academy of Sciences, Beijing 100049, China

(Received 2022.05.02; Revised manuscript received XXXX)

# **Appendix**

In this appendix, we will introduce more about the Fuchsian differential equation and Heun equation.

The Fuchsian equation is a linear homogeneous ordinary differential equation with analytic coefficients whose singular points are all regular singular points on the complex plane. The well-known Hilbert’s 21st problem is to find a Fuchsian equation with prescribed singularities and prescribed monodromies. One specific expression of the Fuchsian equation is [46]:

|  | $y^{(n)}(z)+p_{1}(z)y^{(n-1)}(z)+p_{2}(z)y^{(n-2)}(z)+\ldots+p_{n}(z)y(z)=0$ | (104) |
| --- | --- | --- |

Where:

|  | $p_{j}(z)=\frac{q_{j}(z)}{\prod_{m=1}^{k} \left(z-z_{m}\right)^{j}}$ | (105) |
| --- | --- | --- |

And $S=\left\{z_{1},...,z_{k}\right\}$ are regular singular points at which solutions have local monodromies and $q_{j}(z)$ is a polynomial of degree $\leq\text{ }j(k-1)$. For each singularity $z_{m}\in S$, the characteristic exponents are roots of the characteristic equation, which is an algebraic equation of degree *n*. We denote characteristic exponents by $\lambda_{z_{m},1},\lambda_{z_{m},2},\ldots,\lambda_{z_{m},n}$. Then we have Fuchs relation [47]:

|  | $\sum_{z_{m}\in S} \sum_{j=1}^{n} \lambda_{z_{m},j}=\frac{n(n-1)(k-2)}{2}$ | (106) |
| --- | --- | --- |

The differential equation of the standard level-two monodromy method (18) belongs to the Heun equation which is the known Fuchsian equation with four singularities [47, 48]. One famous canonical form of the Heun equation presents as follows:

|  |  | (107) |
| --- | --- | --- |

Obviously, the differential equation has four regular singularities at 0, 1, *a*, ∞. It is necessary to assume the condition $\varepsilon=\alpha+\beta-\gamma-\delta+1$ to ensure the regularity of the point at ∞. We call parameter a the singularity parameter, $\alpha,\text{ }\beta,\text{ }\gamma,\text{ }\delta,\text{ }\varepsilon$, exponent parameters. The remaining quantity $q$, known as the accessory parameter of the equation, involves global monodromy properties of (107). So there are six free parameters in total in the Heun equation. Mathematically, it can be proved that all other homogeneous linear differential equations of the second order, which have four regular singularities in the Riemann sphere, can be transformed into equation (107). Another form of the Heun equation known as the normal form behaves as follows [49]:

|  | $\psi^{''}(z)-\left(\frac{A}{z}+\frac{B}{z-1}+\frac{C}{z-a}+\frac{D}{z^{2}}+\frac{E}{(z-1)^{2}}+\frac{F}{(z-a)^{2}}\right)\psi(z)=0$ | (108) |
| --- | --- | --- |

Above two forms can be linked by the substitution , where:

|  | $\begin{matrix} A=-\frac{\gamma\delta}{2}-\frac{\gamma\varepsilon}{2a}+\frac{q}{a}, B=\frac{\gamma\delta}{2}-\frac{\gamma\varepsilon}{2(a-1)}-\frac{q-\alpha\beta}{a-1}, C=\frac{\gamma\varepsilon}{2}+\frac{\gamma\varepsilon}{2(a-1)}-\frac{a\alpha\beta-q}{a(a-1)} \\ D=\frac{1}{2}\gamma\left(\frac{1}{2}\gamma-1\right), E=\frac{1}{2}\delta\left(\frac{1}{2}\delta-1\right), F=\frac{1}{2}\varepsilon\left(\frac{1}{2}\varepsilon-1\right) \end{matrix}$ | (109) |
| --- | --- | --- |

And the condition $A+B+C=0$. There are also other various forms of the Heun equation such as the trigonometric form, doubly-periodic forms, etc., which we will not discuss here.

A power series solution of equation (107) is the Heun function:

|  | $H(a,q;\alpha,\beta,\gamma,\delta;z)=\sum_{n=0}^{\infty} h_{n}x^{n}$ | (110) |
| --- | --- | --- |

Where $h_{0}=1$ and other expansion coefficients are defined by a recursive relation:

|  | $\begin{matrix} a\gamma h_{1}-qh_{0}=0, R_{n}h_{n+1}-\left(Q_{n}+q\right)h_{n}+P_{n}h_{n-1}=0, n=1,2,\ldots, \\ R_{n}=(n-1+\alpha)(n-1+\beta), \\ Q_{n}=n((n-1+\gamma)(1+\alpha)+a\delta+(\alpha+\beta-\gamma-\delta+1)), \\ P_{n}=a(n+1)(n+\gamma) \end{matrix}$ | (111) |
| --- | --- | --- |

In this paper we are interested in studying a realization of the Fuchsian equation, decoupling equation, in 2d CFT and discussing some of its consequences. In 2d CFT, the differential equation of the standard monodromy method (18) that occurred in the normal form (108), the differential equaion of other-level monodromy method (41) and (88) have been discussed in this paper. Besides the monodromy method, the Fuchsian equation has many other physical applications. For instance, the solution of the problem of adding three quantum spins is given in terms of a product of two Heun functions. Fuchsian equations also appear in the black hole scattering theory [50, 51], lattice systems in statistical mechanics [52, 53], and solutions of the Schrodinger equation [54-56].

# **References**

1. Haraoka, Y. Linear Differential Equations in the Complex Domain (Suugaku Shobou, 2015).
2. Ronveaux, A. & Arscott, F. M. Heun’s Differential Equations (Oxford University Press, 1995).
3. Maier, R. S. The 192 solutions of the Heun equation. Math. Comput. 76(258), 811–843 (2006).
4. Piatek, M. & Pietrykowski, A. R. Solving Heun’s equation using conformal blocks. Nuclear Phys. B 938, 543–570 (2019).
5. Suzuki, H., Takasugi, E. & Umetsu, H. Perturbations of Kerr-de Sitter black holes and Heun’s equations. Prog. Theoret. Phys. 100(3), 491–505 (1998).
6. Kalnins, E. G., Miller, W. Jr., TorresdelCastillo, G. F. & Williams, G. C. Special functions and perturbations of black holes. In Special functions (Hong Kong, 1999) (eds Dunkl, C. F. et al.) 140–151 (World Sci. Publishing, 2000).
7. Joyce, G. S. On the simple cubic lattice Green function. Philos. Trans. R. Soc. Lond. Ser. A 273, 583–610 (1973).
8. Joyce, G. S. On the cubic lattice Green functions. Proc. R. Soc. Lond. Ser. A 445, 463–477 (1994).
9. Bay, K., Lay, W. & Akopyan, A. Avoided crossings of the quartic oscillator. J. Phys. A 30(9), 3057–3067 (1997).
10. Tolstikhin, O. I. & Matsuzawa, M. Hyperspherical elliptic harmonics and their relation to the Heun equation. Phys. Rev. A 63(032510), 1–8 (2001).
11. Hall, R. L., Saad, N. & Sen, K. D. Soft-core Coulomb potentials and Heun’s differential equation. J. Math. Phys. 51(2), 022107 (2010).

1. *Corresponding Author, E-mail: houyuanpeng@xao.ac.cn [↑](#footnote-ref-0)
